# Mechanical Versus Kinematic Alignment in Total Knee Arthroplasty: Relationship Between Postoperative Alignment and Short-Term Functional Outcomes

**DOI:** 10.3390/diagnostics16213446

**Published:** 2026-10-22

**Authors:** Giada Baroni, Elena Lucchesini, Dan Fruja, Ioana Teodora Floarea, Ana-Liana Bouroș-Tataru, Laura Ioana Rădoi, Nicoleta Anamaria Pascalau, Corina Dalia Toderescu, Georgeta Lucia Piscoi, Alexandru Pop

**Affiliations:** 1 Doctoral School of Medicine, “Vasile Goldiș” Western University of Arad, 310025 Arad, Romania; baroni.giada@student.uvvg.ro (G.B.); lucchesini.elena@student.uvvg.ro (E.L.); pop.alexandru@uvvg.ro (A.P.); 2 Department of General Medicine, Faculty of Medicine, “Vasile Goldiș” Western University of Arad, 310025 Arad, Romania; fruja.dan@uvvg.ro (D.F.); floarea.ioana@uvvg.ro (I.T.F.); 3 Doctoral School of Medicine, “Victor Babeș” University of Medicine and Pharmacy, 300041 Timișoara, Romania; 4 Department of Biology and Life Sciences, Faculty of Medicine, “Vasile Goldiș” Western University of Arad, 310025 Arad, Romania; 5 Department of Psycho Neuroscience and Recovery, Faculty of Medicine and Pharmacy, University of Oradea, 410087 Oradea, Romania; nicoleta.pascalau@didactic.uoradea.ro; 6 Faculty of Pharmacy, “Vasile Goldiș” Western University of Arad, 310045 Arad, Romania; toderescu.corina@uvvg.ro; 7 Faculty of Physical Education and Sport, “Aurel Vlaicu” University of Arad, 310130 Arad, Romania; georgeta.piscoi@uav.ro

**Keywords:** arthroplasty, kinematic alignment, knee, mechanical alignment, osteoarthritis, radiography, treatment outcome

## Abstract

**Background/Objectives:** Mechanical alignment (MA) has traditionally been the reference alignment strategy in total knee arthroplasty (TKA), whereas kinematic alignment (KA) has emerged as an individualized alternative intended to approximate patient-specific knee anatomy. However, the relationship between postoperative mechanical-axis alignment and functional recovery remains incompletely understood. This study aimed to compare short-term functional outcomes and postoperative mechanical-axis alignment between MA and KA following primary TKA and to investigate the relationship between measured postoperative alignment parameters and functional outcomes. **Methods:** This single-center retrospective comparative cohort study included 120 patients who underwent primary unilateral TKA for end-stage knee osteoarthritis (KOA) between January 2022 and December 2024; 51 underwent MA and 69 underwent KA. Radiographic assessment included the hip–knee–ankle (HKA) angle, mechanical-axis deviation (MAD), residual varus deformity, and radiographic knee extension deficit. Functional outcomes were assessed using the Knee injury and Osteoarthritis Outcome Score (KOOS), the Western Ontario and McMaster Universities Osteoarthritis Index (WOMAC), and the Knee Society Score (KSS). Preoperative, postoperative, and change-from-baseline (Δ) functional scores were evaluated. Analysis of covariance (ANCOVA) was used to assess associations between alignment strategy and postoperative functional outcomes after adjustment for baseline functional scores and relevant covariates. Associations between postoperative radiographic parameters and functional outcomes were additionally assessed. **Results:** Both alignment groups demonstrated substantial preoperative-to-postoperative functional improvement. MA achieved postoperative HKA alignment closer to the neutral mechanical axis than KA (179.63 ± 1.23° vs. 178.24 ± 2.32°; *p* < 0.001; FDR-adjusted *p* < 0.001). Absolute HKA deviation from neutral was lower with MA (0.97 ± 0.83° vs. 2.42 ± 1.62°; FDR-adjusted *p* < 0.001), as was postoperative absolute MAD (5.19 ± 4.53 mm vs. 7.86 ± 6.76 mm; *p* = 0.011; FDR-adjusted *p* = 0.018). KA was associated with a higher unadjusted postoperative KOOS score than MA (84.04 ± 12.33 vs. 77.12 ± 15.65; *p* = 0.010), whereas postoperative WOMAC (*p* = 0.206) and KSS (*p* = 0.843) did not differ significantly between groups. Between-group differences in change from baseline were not statistically significant for KOOS (*p* = 0.227), WOMAC (*p* = 0.130), or KSS (*p* = 0.427). After multivariable adjustment, no statistically significant between-group associations were observed for postoperative KOOS (adjusted β = 2.42; 95% CI, −0.93 to 5.77; *p* = 0.155), WOMAC (adjusted β = −2.80; 95% CI, −6.73 to 1.14; *p* = 0.162), or KSS (adjusted β = 1.09; 95% CI, −2.05 to 4.24; *p* = 0.492). Associations between the postoperative radiographic parameters and functional outcomes were negligible to weak, with none remaining statistically significant after FDR correction. **Conclusions:** Both MA and KA were associated with substantial short-term functional improvement, with no significant between-group differences in the magnitude of improvement from baseline. MA achieved postoperative alignment closer to the neutral mechanical axis, while the higher unadjusted postoperative KOOS score observed with KA was not statistically significant after adjustment for baseline characteristics and potential confounders. No significant postoperative differences in WOMAC or KSS were observed. The generally weak associations between measured postoperative mechanical-axis parameters and functional outcomes suggest that mechanical-axis alignment alone may not adequately explain short-term functional recovery. Because patient-specific coronal alignment parameters and achievement of the individualized KA target were not quantitatively assessed, the present findings cannot determine whether restoration of patient-specific anatomy was associated with functional outcomes.

## 1. Introduction

Knee osteoarthritis (KOA) is a leading cause of pain, disability, and reduced quality of life worldwide and represents a growing public health burden in the context of population aging and the increasing prevalence of obesity [1,2,3]. KOA is increasingly recognized as a progressive whole-joint disorder in which mechanical, inflammatory, metabolic, and molecular mechanisms interact to drive structural degeneration and functional impairment. Progressive articular cartilage degeneration, subchondral bone remodeling, and synovial inflammation contribute to pain, deformity, functional limitation, and loss of mobility [4,5,6]. Alterations in cartilage structure and biomechanical properties further compromise load distribution, shock absorption, and low-friction articulation, contributing to progressive joint dysfunction [7,8]. When conservative treatment fails to provide adequate symptom relief in patients with end-stage disease, total knee arthroplasty (TKA) is an established treatment that provides substantial pain relief, functional improvement, and durable implant survival [9,10,11].

Traditionally, TKA has been performed using mechanical alignment (MA), which aims to position the femoral and tibial components perpendicular to their respective mechanical axes and restore a neutral hip–knee–ankle (HKA) angle of approximately 180° [12,13,14]. MA has long been regarded as the reference alignment strategy because neutral MA has been proposed to optimize load distribution across the prosthesis, reduce polyethylene wear, and support implant longevity [15,16,17]. However, increasing recognition of substantial interindividual variability in constitutional limb alignment and native joint-line orientation has challenged the concept that a uniform neutral alignment target is necessarily optimal for every patient. This has contributed to increasing interest in individualized alignment strategies that seek to preserve patient-specific anatomy and knee kinematics while maintaining implant stability.

Despite the established effectiveness and long-term survivorship of TKA, approximately 15–20% of patients report persistent pain, functional limitations, or dissatisfaction following surgery [18,19,20]. Postoperative dissatisfaction is multifactorial and may reflect residual pain, stiffness, instability, altered knee kinematics, soft-tissue imbalance, and differences between patient expectations and postoperative function. These observations have contributed to interest in whether individualized component positioning and preservation of native knee anatomy may improve functional recovery compared with systematic restoration of a neutral mechanical axis.

Among individualized alignment strategies, kinematic alignment (KA) has emerged as one of the most extensively investigated alternatives to MA. Rather than systematically restoring a neutral mechanical axis, KA seeks to reproduce the patient’s pre-arthritic joint-line orientation and constitutional limb alignment while preserving native ligament balance and minimizing unnecessary soft-tissue releases [21,22,23]. It has been proposed that this approach may more closely reproduce physiological knee kinematics and thereby improve patient satisfaction and functional recovery while maintaining acceptable implant-related outcomes [24,25,26]. However, the optimal balance between mechanical-axis alignment, implant biomechanics, and patient-specific functional restoration remains debated. Importantly, postoperative alignment relative to the neutral mechanical axis does not, by itself, indicate whether the intended patient-specific alignment target has been achieved.

Although randomized controlled trials and systematic reviews have compared MA and KA, the superiority of either alignment strategy remains uncertain. Some studies have reported improved patient-reported outcomes following KA, including higher Knee injury and Osteoarthritis Outcome Score (KOOS), Knee Society Score (KSS), and Forgotten Joint Score, whereas others have found comparable clinical outcomes despite differences in postoperative alignment [27,28,29]. Concerns have also been raised regarding the potential long-term consequences of alignment outside the neutral mechanical-axis range for implant fixation, polyethylene wear, and survivorship, although available mid-term studies have not consistently demonstrated statistically significant differences in revision rates between MA and KA [30,31].

Importantly, the relationship between postoperative mechanical-axis alignment and functional recovery remains incompletely understood. Differences in postoperative HKA or mechanical-axis deviation (MAD) between alignment strategies do not necessarily imply corresponding differences in patient-reported or clinical outcomes. Furthermore, studies directly examining associations between measured postoperative mechanical-axis parameters and functional outcomes within the same cohort remain relatively limited. Clarifying this relationship may help determine the extent to which postoperative alignment relative to the neutral mechanical axis accounts for variation in short-term functional recovery following TKA.

The aim of the present retrospective cohort study was therefore to compare short-term functional outcomes and postoperative mechanical-axis alignment between MA and KA following primary TKA and to investigate the relationship between measured postoperative alignment parameters and functional outcomes. The primary clinical outcomes were postoperative KOOS, Western Ontario and McMaster Universities Osteoarthritis Index (WOMAC), and KSS. Secondary analyses evaluated postoperative radiographic parameters, changes in functional outcomes from baseline, and the magnitude of postoperative deviation from the neutral mechanical axis, without treating this deviation as a measure of KA target attainment. Associations between postoperative radiographic parameters and functional outcomes were additionally investigated, while complications and reoperations observed during the available follow-up were summarized descriptively.

It was hypothesized that both alignment strategies would be associated with substantial short-term functional improvement, while MA would result in postoperative mechanical-axis alignment closer to neutral. We further hypothesized that associations between measured postoperative mechanical-axis parameters and functional outcomes would be limited, reflecting the multifactorial nature of functional recovery following TKA.

## 2. Materials and Methods

### 2.1. Study Design

This retrospective comparative cohort study was conducted at the Clinical Department of Orthopaedics and Traumatology, Arad County Emergency Clinical Hospital, Arad, Romania. Medical records of patients evaluated for eligibility following knee arthroplasty performed between January 2022 and December 2024 were retrospectively reviewed. The final study cohort comprised patients who underwent primary unilateral TKA for end-stage KOA. Patients were classified according to the alignment strategy used during surgery as MA or KA. Given the available follow-up period, the study focused on short-term radiographic and functional outcomes and was not designed to evaluate long-term implant survivorship or comparative revision risk.

Clinical records, operative reports, radiographs, and functional outcome data were retrospectively analyzed to compare postoperative functional outcomes and mechanical-axis alignment between MA and KA and to investigate the relationship between measured postoperative radiographic parameters and functional outcomes. Available follow-up records were additionally reviewed to descriptively identify postoperative complications, reoperations, aseptic loosening, and revision procedures occurring during the observation period.

The primary clinical outcomes were postoperative KOOS, WOMAC, and KSS. Secondary outcomes included postoperative radiographic parameters, changes in functional outcomes from baseline, and postoperative alignment measured relative to the neutral mechanical axis rather than achievement of an individualized KA target. Additional exploratory analyses evaluated correlations between postoperative radiographic parameters and functional outcomes. Postoperative complications and reoperations identified during the available follow-up were assessed descriptively because the study was not designed or powered for comparative safety or survivorship analyses.

The study was conducted and reported in accordance with the Strengthening the Reporting of Observational Studies in Epidemiology (STROBE) guidelines for observational cohort studies.

### 2.2. Patient Selection

Medical records of 158 consecutive patients who underwent knee arthroplasty between January 2022 and December 2024 were screened for eligibility. Patients were selected according to predefined inclusion and exclusion criteria. Because of the retrospective design, no a priori sample-size calculation was performed; the study sample comprised all consecutive eligible patients meeting the inclusion and exclusion criteria during the study period.

Patients were eligible for inclusion if they met all of the following criteria:

- Age ≥ 50 years;
- Diagnosis of primary end-stage KOA;
- Kellgren–Lawrence (KL) grade III or IV;
- Primary unilateral TKA performed using either MA or KA;
- Availability of complete preoperative and postoperative radiographic examinations;
- Availability of complete preoperative and postoperative functional outcome assessments (KOOS, WOMAC, and KSS);
- Minimum postoperative follow-up of 12 months.

Patients were excluded if any of the following criteria were present:

- Revision TKA;
- Inflammatory arthritis (e.g., rheumatoid arthritis);
- Previous osteotomy or fracture around the affected knee;
- Incomplete clinical or radiographic data;
- Loss to follow-up before the minimum required postoperative assessment.

**Figure 1:**
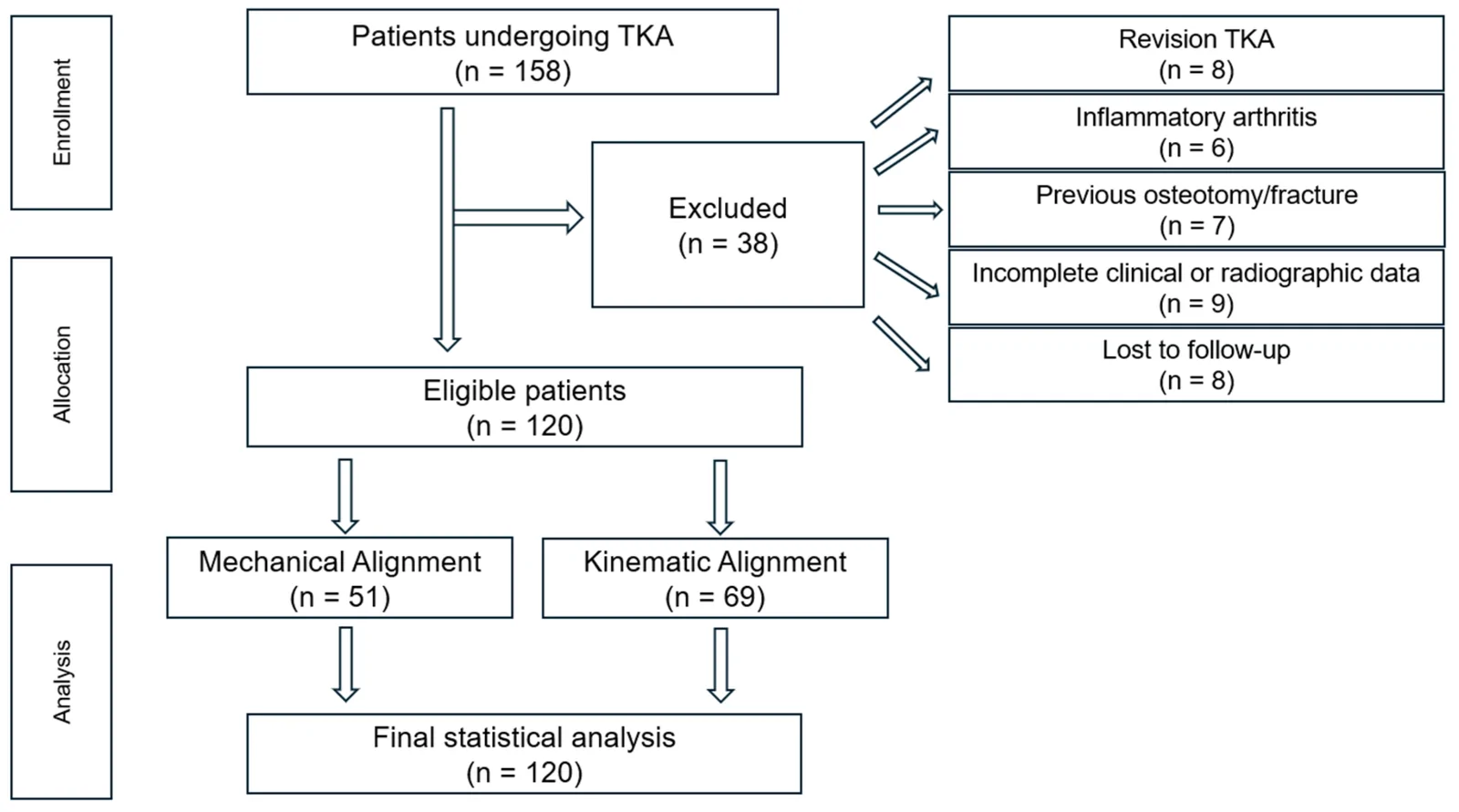
Flowchart of patient selection and classification according to alignment strategy.

After application of the eligibility criteria, 120 patients were included in the final analysis, comprising 51 patients treated using MA and 69 patients treated using KA (Figure 1).

The final analytical cohort was restricted to patients with complete preoperative and postoperative clinical, radiographic, and functional outcome data and the required minimum follow-up; consequently, patients excluded because of incomplete data or insufficient follow-up could not contribute to the comparative analyses, potentially introducing selection bias.

### 2.3. Surgical Technique

All procedures were performed by three experienced orthopedic surgeons at the Clinical Department of Orthopaedics and Traumatology, Arad County Emergency Clinical Hospital, using a standardized medial parapatellar approach under tourniquet control. Conventional, implant-specific instrumentation was used without robotic assistance or computer-assisted navigation. All patients received a cemented primary TKA using the Triathlon^®^ Total Knee System (Stryker Orthopaedics, Mahwah, NJ, USA), exclusively in a posterior-stabilized (PS) configuration. Both the anterior cruciate ligament (ACL) and posterior cruciate ligament (PCL) were sacrificed in all cases. The collateral ligaments and extensor mechanism were protected throughout the procedure. Patellar resurfacing was performed selectively according to the surgeon’s intraoperative assessment of patellar cartilage degeneration and patellofemoral tracking.

Treatment allocation was not randomized. All three participating surgeons performed both MA and KA procedures, and both strategies were used throughout the study period. The choice of alignment strategy was based on the operating surgeon’s clinical judgment, taking into account patient-specific clinical characteristics and the severity of articular degeneration. According to the surgical team’s estimates, MA had been used in the department for approximately 15 years, whereas KA had been introduced approximately 1 year before the start of the study period; however, the exact dates of introduction were not independently verified. The number of MA and KA procedures performed by each individual surgeon within the study cohort was not quantified. Patients were classified retrospectively according to the alignment strategy documented in the operative records and the corresponding surgical approach.

In the MA group, the surgical objective was restoration of a neutral lower-limb mechanical axis, corresponding to an HKA angle of approximately 180°. Conventional mechanical-alignment cutting guides were used to orient the femoral and tibial components relative to their respective mechanical axes in the coronal plane.

In the KA group, component positioning was individualized according to the patient’s preoperative radiographic anatomy, with particular emphasis on reproducing the planned tibial joint-line orientation and constitutional alignment. The presumed pre-arthritic joint-line orientation was estimated from preoperative radiographs and intraoperative anatomical landmarks, including relatively preserved articular surfaces, while accounting for cartilage loss and degenerative changes in the affected compartments. Unlike the standardized mechanical-axis references used in MA, tibial component alignment was adapted to the patient’s anatomical characteristics and preoperative radiographic assessment. The intended global coronal alignment was limited to within ±3° of neutral. The predefined ±3° coronal alignment range represented an intended surgical target rather than a guaranteed postoperative result. Postoperative deviations beyond this range may therefore reflect discrepancies between planned and achieved alignment; however, individual target achievement could not be independently verified because patient-specific alignment targets were not systematically documented. Femoral component rotation was standardized at 3° of external rotation relative to the posterior condylar reference in both groups, with the transepicondylar axis used as an additional anatomical reference. Accordingly, the approach used in the KA group is best characterized as a modified, constrained kinematic-alignment technique, incorporating individualized tibial alignment within predefined global coronal alignment limits and a standardized femoral rotational setting, rather than unrestricted KA.

In both groups, bone resections were performed using conventional adjustable cutting guides, with femoral preparation preceding tibial preparation. Femoral resection levels were established using anatomical references and the condition of the articular surfaces. In regions with preserved articular cartilage, an 8 mm resection reference was used. In areas with severe cartilage loss and exposed or damaged subchondral bone, approximately 2 mm of subchondral bone was resected to obtain a suitable bone surface for cemented component fixation. Resection thickness was determined using the cutting guides without additional caliper verification, and saw-blade thickness was not separately incorporated into the resection calculations.

A neutral tibial slope (0°) was generally targeted in both alignment groups. In selected cases involving a stiff or locked knee, approximately 2° of anterior tibial slope was used according to intraoperative judgment. This exception was uncommon, but its exact frequency within the study cohort was not recorded.

Following completion of the bone resections, trial components were inserted. Component position, limb alignment, flexion and extension stability, range of motion, soft-tissue balance, and patellar tracking were assessed intraoperatively. Final ligament balance was evaluated after component trialing, and additional bone-resection adjustments or selective soft-tissue releases were performed when considered necessary to achieve satisfactory stability. In the KA group, unnecessary ligament releases were avoided where possible, with the aim of preserving functional soft-tissue balance.

After satisfactory trial assessment, definitive Triathlon^®^ PS components were implanted using Simplex^®^ P bone cement (Stryker Orthopaedics, Mahwah, NJ, USA). Knee stability, alignment, range of motion, and patellar tracking were reassessed before wound closure. Standard postoperative rehabilitation, including early mobilization and progressive weight-bearing as tolerated, was applied to all patients.

The retrospective dataset did not permit systematic quantitative assessment of achievement of the individualized KA target. Furthermore, medial proximal tibial angle (mPTA), lateral distal femoral angle (LDFA), joint-line obliquity (JLO), and coronal plane alignment of the knee (CPAK) phenotype were not systematically measured or recorded in the original dataset. Therefore, postoperative HKA and mechanical axis deviation (MAD) were used to characterize alignment relative to the neutral mechanical axis and should not be interpreted as quantitative measures of accuracy in achieving the individualized KA target or restoring the native coronal phenotype. Although the surgical team reported following a common set of technical principles, patient-specific planned component angles and adherence to these targets were not systematically documented. Consequently, the consistency of implementation and target achievement across individual KA procedures could not be independently verified.

**Figure 2:**
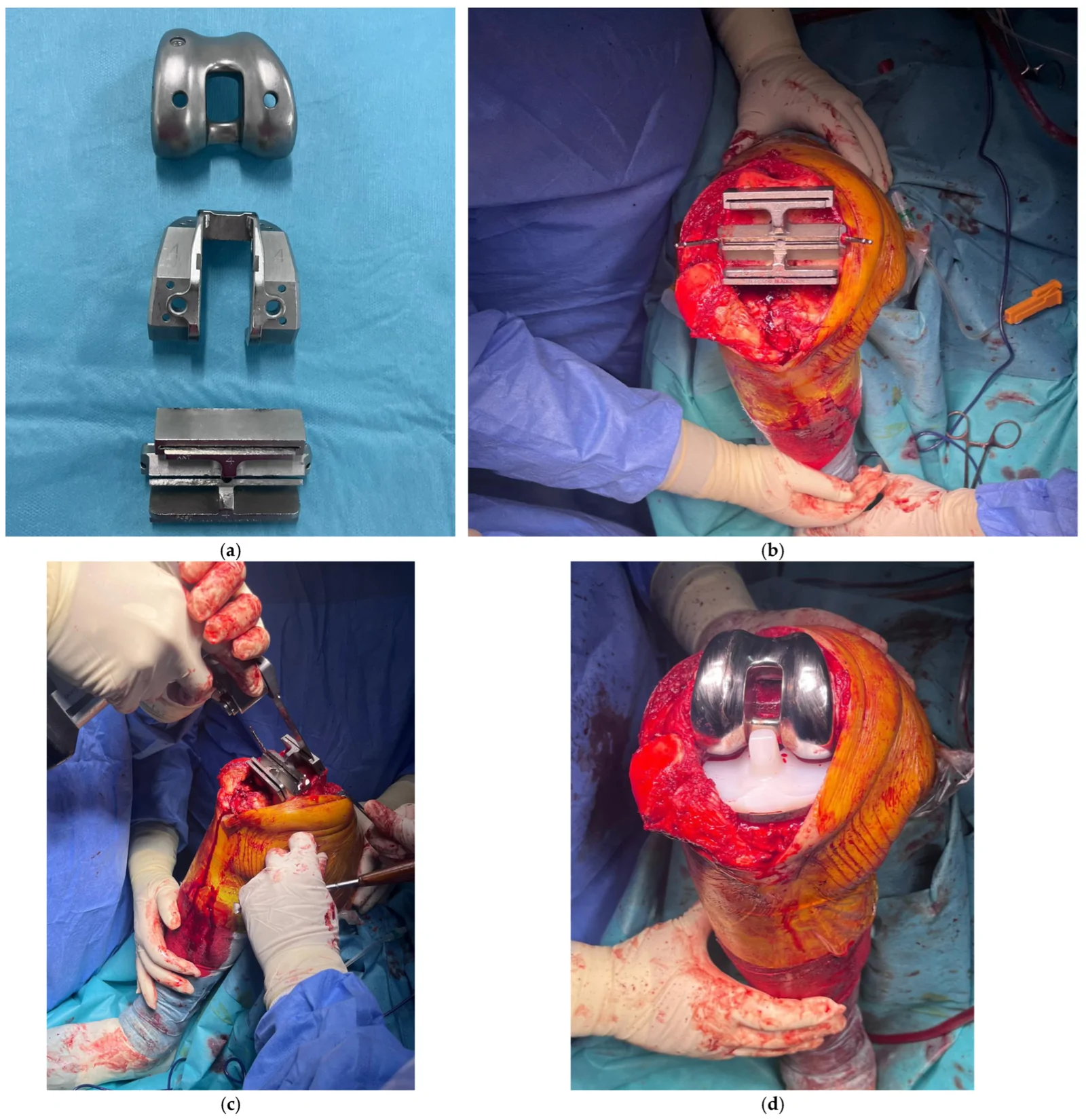
Representative intraoperative steps during primary TKA. (**a**) Representative cutting instrumentation used during the procedure. (**b**) Intraoperative positioning of the cutting instrumentation. (**c**) Bone resection performed using an oscillating saw with the cutting instrumentation in position. (**d**) Intraoperative appearance following implantation of the definitive femoral and tibial components. The images illustrate general procedural steps and are not intended to demonstrate the technical distinctions between MA and KA.

Representative intraoperative steps are illustrated in Figure 2.

### 2.4. Radiographic Evaluation

Radiographic assessment was performed using standardized full-length standing anteroposterior radiographs of the lower extremities together with lateral knee radiographs obtained preoperatively and at the final postoperative follow-up. Full-length anteroposterior radiographs were acquired according to the institutional standardized imaging protocol, with patients standing in full weight-bearing and the patellae oriented anteriorly to minimize rotational error. Lateral knee radiographs were obtained with patients in the supine position and instructed to extend the operated knee as fully as possible.

The radiographic parameters evaluated were the HKA angle, MAD, residual varus deformity, and radiographic knee extension deficit. The HKA angle was defined as the angle formed by the mechanical axes of the femur and tibia, with 180° representing neutral MA. MAD was measured as the perpendicular distance, in millimeters, between the lower-limb mechanical axis and the center of the tibial plateau and was recorded as a signed value, with negative values indicating medial displacement (varus) and positive values indicating lateral displacement (valgus). Residual varus deformity was assessed on anteroposterior radiographs as an angular measurement based on the anatomical axes of the femur and tibia. The anatomical axes were defined according to the longitudinal orientation of the femoral and tibial shafts. This measurement was recorded in degrees and evaluated separately from the HKA angle, which was based on the mechanical axes of the lower limb. Residual varus deformity was therefore not calculated as the arithmetic difference between 180° and the HKA angle. The two parameters were retained to characterize coronal alignment using different reference axes, rather than as independent measures of unrelated anatomical phenomena.

The radiographic knee extension deficit was measured as the angular deviation from full extension on lateral knee radiographs. This measurement represented the degree of incomplete extension observed during radiographic acquisition and was not considered equivalent to a clinically confirmed fixed flexion contracture.

Radiographic measurements were independently performed by two orthopedic surgeons who were blinded to the patients’ functional outcome scores. Interobserver reliability was assessed by independent measurements of HKA, MAD, residual varus deformity, and radiographic knee extension deficit by both observers in a randomly selected subset of 30 patients. For intraobserver reliability, the first observer repeated the measurements after an interval of 4 weeks without access to the initial measurements. Intra- and interobserver reliability were quantified using intraclass correlation coefficients (ICCs) with corresponding 95% confidence intervals (CIs). Reliability was evaluated using a two-way absolute-agreement, single-measurement ICC model ICC(A,1), with 95% CIs estimated by patient-level bootstrap resampling (5000 iterations).

An HKA angle within ±3° of neutral (180°) and an absolute postoperative MAD (|MAD|) < 5 mm were recorded as descriptive measures of postoperative mechanical-axis alignment. HKA deviations > 3° and >5° from neutral were additionally recorded. Absolute HKA deviation was calculated as |HKA − 180°|, while |MAD| represented the magnitude of mechanical-axis displacement irrespective of direction. In the MA group, these measures were directly relevant to the intended objective of neutral MA. In the KA group, however, deviation from 180° was interpreted only as deviation from the neutral mechanical axis and not as evidence of technical error or failure to achieve the individualized KA target.

The available radiographic dataset did not permit comprehensive assessment of patient-specific coronal alignment following KA. Although preoperative and postoperative standing long-leg radiographs were available for all included patients, mPTA, LDFA, and JLO were not systematically measured or recorded during the original retrospective data extraction and analysis. Consequently, these parameters and the derived CPAK phenotype were not included in the present analyses. Their absence from the analytical dataset should not be interpreted as evidence that they cannot be measured retrospectively from the archived radiographs. Moreover, achievement of the individualized KA target was not quantitatively assessed. Consequently, postoperative HKA and MAD in the KA group should be interpreted as descriptors of alignment relative to the neutral mechanical axis rather than as measures of successful restoration of the native coronal phenotype or technical accuracy in achieving the intended KA alignment.

Postoperative radiographic measurements were compared between the MA and KA groups to characterize differences in mechanical-axis alignment. Postoperative radiographic parameters were also correlated with KOOS, WOMAC, and KSS to investigate the relationship between measured postoperative alignment and functional outcomes.

### 2.5. Clinical Outcome Assessment

Clinical outcomes were assessed preoperatively and at the final postoperative follow-up using the KOOS, WOMAC, and KSS. These measures provide complementary assessments of pain, symptoms, physical function, and knee-related clinical status following TKA.

KOOS is a patient-reported outcome measure comprising five domains: pain, symptoms, activities of daily living, sport and recreation, and knee-related quality of life. Each domain was scored from 0 to 100, with higher scores indicating better knee-related health status. For the present analysis, a study-defined composite KOOS score was calculated as the unweighted arithmetic mean of the five domain scores. The composite score was used consistently for preoperative and postoperative assessments. It was intended to provide a single descriptive summary of knee-related symptoms, function, and quality of life for the main between-group comparisons, rather than to serve as a separately validated standard KOOS total score. Domain-specific preoperative and postoperative results for both alignment groups are presented in Supplementary Materials Table S1.

WOMAC assesses pain, stiffness, and physical function. The 24-item Likert version was used, comprising pain (5 items), stiffness (2 items), and physical function (17 items). Each item was scored from 0 to 4, yielding a total score ranging from 0 to 96, with lower scores indicating less pain and stiffness and better physical function.

The original Knee Society Scoring System was used, and the clinical knee score (0–100 points) was recorded for analysis. This component evaluates pain, range of motion, and stability, with deductions for flexion contracture, extension lag, and malalignment. The separate functional score was not included in the present analysis. Unlike the patient-reported KOOS and WOMAC instruments, the clinical KSS component represents a clinician-assessed measure of objective knee status and should not be interpreted as a separate patient-reported functional outcome. Higher scores indicated better clinical knee status.

Assessments were performed during routine outpatient follow-up using standardized evaluation procedures. The KOOS and WOMAC questionnaires were administered in Romanian and completed by patients with assistance from medical staff when needed. All questionnaires included in the analysis had complete item-level responses; therefore, no imputation of missing questionnaire items was required. KOOS, WOMAC, and KSS were recorded preoperatively and at the final postoperative assessment. Clinical assessments were performed by experienced orthopedic clinicians who were not involved in the radiographic measurements. Because the clinical KSS incorporates alignment-related characteristics and extension deficits, correlations between KSS and postoperative radiographic parameters were considered potentially affected by mathematical overlap and were therefore interpreted as exploratory.

The primary clinical outcomes were postoperative KOOS, WOMAC, and KSS scores compared between the MA and KA groups. Changes from baseline to final postoperative follow-up (Δ scores) were additionally calculated for each measure to compare the magnitude of functional improvement between alignment strategies. For all three measures, change scores were calculated as the postoperative score minus the preoperative score; positive changes indicated improvement for KOOS and KSS, whereas negative changes indicated improvement for WOMAC.

### 2.6. Follow-Up and Postoperative Complications

Postoperative follow-up duration was calculated from the date of primary TKA to the most recent documented clinical evaluation available in the medical record. Available clinical records, operative reports, subsequent hospitalization records, and follow-up documentation were retrospectively reviewed to identify postoperative complications and subsequent surgical procedures involving the operated knee during the observation period.

The postoperative events recorded were periprosthetic joint infection (PJI), reoperation for any reason, aseptic loosening, and revision TKA. Reoperation was defined as any subsequent surgical procedure involving the operated knee, including procedures involving prosthetic component removal, exchange, or replacement, whereas revision TKA was defined as removal, exchange, or replacement of one or more prosthetic components. Thus, revision TKA was considered a subset of reoperations rather than an independent, mutually exclusive event category. PJI and aseptic loosening were recorded when documented in the available medical records on the basis of the treating team’s clinical, laboratory, imaging, and/or operative assessment, as applicable. Events were identified from the available retrospective documentation, without assuming that complications managed outside the reporting institution were systematically captured.

Because of the small number of events and the limited duration of follow-up, postoperative complications and reoperations were summarized descriptively without inferential between-group comparisons. These data were intended to characterize events observed during the available follow-up period and were not used to evaluate comparative long-term safety, implant survivorship, or revision risk between MA and KA. The absence of a statistically tested difference in complication rates should therefore not be interpreted as evidence of equivalent safety between the two alignment strategies.

### 2.7. Statistical Analysis

Statistical analyses were performed using IBM SPSS Statistics (Version 29.0; IBM Corp., Armonk, NY, USA). Continuous variables were assessed for normality using the Shapiro–Wilk test before statistical analysis. Normally distributed continuous variables are presented as mean ± standard deviation (SD), whereas categorical variables are presented as frequencies and percentages.

Comparisons of baseline characteristics and postoperative outcomes between the MA and KA groups were performed using Welch’s independent-samples *t*-tests for continuous variables and the χ^2^ test or Fisher’s exact test, as appropriate, for categorical variables. For continuous between-group comparisons, mean differences were calculated as KA − MA and are reported with corresponding 95% CIs. Standardized between-group differences were additionally quantified using Cohen’s d. Effect-size magnitudes were interpreted as small (0.20–0.49), medium (0.50–0.79), and large (≥0.80).

Follow-up duration was summarized descriptively for the overall cohort and separately for the MA and KA groups. Postoperative complications, including PJI, reoperation, aseptic loosening, and revision TKA, were reported as frequencies and percentages. Because of the small number of events and limited follow-up, no inferential between-group comparisons were performed for complication or reoperation rates.

Preoperative-to-postoperative changes in radiographic and functional outcome measures were evaluated using paired-samples *t*-tests. For KOOS, WOMAC, and KSS, individual change scores (Δ) were calculated as the postoperative value minus the corresponding preoperative value. Accordingly, positive Δ values for KOOS and KSS and negative Δ values for WOMAC indicated functional improvement. Between-group differences in Δ scores were compared using Welch’s independent-samples *t*-tests and are reported as mean differences (KA − MA) with corresponding 95% CIs and Cohen’s d. The magnitude of preoperative-to-postoperative change within the overall cohort was additionally quantified using Cohen’s dz, defined as the mean of the within-patient change scores divided by their SD. For descriptive interpretation, the absolute magnitude of Cohen’s dz was reported.

Intra- and interobserver reliability of radiographic measurements was assessed using ICCs based on a two-way random-effects model with absolute agreement and corresponding 95% CIs. A single-measurement, absolute-agreement ICC model ICC(A,1) was used. Interobserver reliability was assessed using measurements from both observers, whereas intraobserver reliability was assessed using repeated measurements by the first observer after a four-week interval. The 95% CIs were estimated using patient-level bootstrap resampling (5000 iterations). ICC values were interpreted as poor (<0.50), moderate (0.50–0.75), good (0.75–0.90), or excellent (>0.90).

To account for potential confounding in comparisons of postoperative functional outcomes between alignment strategies, separate analysis of covariance (ANCOVA) models were constructed for postoperative KOOS, WOMAC, and KSS. Alignment strategy (MA vs. KA) was entered as the main independent variable, and each model was adjusted for the corresponding preoperative functional score, age, sex, body mass index (BMI), KL grade, and preoperative HKA angle. Adjusted between-group effects were expressed as regression coefficients (*β*) with corresponding 95% CIs and *p*-values. The adjusted coefficients were interpreted as associations rather than causal treatment effects because treatment allocation was not randomized and residual confounding could not be excluded.

Associations between postoperative radiographic parameters and functional outcomes were assessed using Pearson’s correlation coefficient (r). The absolute magnitude of the correlation coefficient (|*r*|) was interpreted as negligible (<0.20), weak (0.20–0.39), moderate (0.40–0.59), strong (0.60–0.79), or very strong (≥0.80). Correlation coefficients for the additional absolute-deviation analyses are reported with 95% CIs derived using Fisher’s z transformation.

To further characterize postoperative alignment relative to the neutral mechanical axis, absolute HKA deviation was calculated as |postoperative HKA − 180°|. Because MAD was recorded as a signed measure of mechanical-axis displacement, |MAD| was used to represent the absolute magnitude of MAD irrespective of direction. Higher values of |HKA − 180°| and |MAD| therefore represented greater deviation from neutral MA. Associations of these absolute-deviation measures with postoperative KOOS, WOMAC, and KSS were evaluated using Pearson’s correlation coefficients. Secondary exploratory analyses assessed their associations with change-from-baseline scores (ΔKOOS, ΔWOMAC, and ΔKSS). These correlations were evaluated in the overall cohort and were additionally explored separately within the MA and KA groups. These measures described deviation from the neutral mechanical axis and were not used to assess achievement of the individualized KA target.

To account for multiple comparisons within secondary and exploratory analyses, the Benjamini–Hochberg false discovery rate (FDR) procedure was applied separately to predefined families of related comparisons: (1) five postoperative radiographic comparisons between MA and KA (HKA, absolute HKA deviation, absolute MAD, residual varus deformity, and radiographic knee extension deficit); (2) three between-group comparisons of change-from-baseline functional outcomes; (3) three comparisons of postoperative alignment relative to the neutral mechanical axis (HKA within ±3° of neutral, |MAD| < 5 mm, and HKA deviation > 5° from neutral); (4) 12 correlations between the four original postoperative radiographic parameters and the three postoperative functional outcomes; (5) six correlations between absolute deviation measures (|HKA − 180°| and |MAD|) and postoperative KOOS, WOMAC, and KSS; and (6) six correlations between the absolute deviation measures and change-from-baseline functional outcomes. Stratified correlations within the MA and KA groups were considered exploratory and were interpreted primarily according to their magnitude and 95% CIs rather than statistical significance. The FDR procedure was applied within each family of comparisons, rather than across all secondary and exploratory analyses combined.

Both unadjusted and FDR-adjusted *p*-values are reported where applicable. The primary comparisons of postoperative KOOS, WOMAC, and KSS between alignment strategies were analyzed separately and were not subjected to FDR correction. Only patients with complete clinical, radiographic, and functional data were included in the statistical analyses; therefore, no imputation of missing data was performed. Because analyses were restricted to complete cases, selection bias related to missing data or incomplete follow-up could not be excluded. All statistical tests were two-tailed. A *p*-value <0.05 was considered statistically significant for primary analyses. For secondary and exploratory analyses subjected to multiple-comparison correction, statistical significance was defined as an FDR-adjusted *p*-value <0.05.

### 2.8. Ethical Considerations

The study was conducted in accordance with the ethical principles of the Declaration of Helsinki and applicable national regulations. The study protocol was reviewed and approved by the Ethics Committee of Arad County Emergency Clinical Hospital, Arad, Romania (Approval No. 81/1/1, approval date 06 June 2024).

Given the retrospective design and the use of anonymized clinical, radiographic, and functional data collected during routine clinical care, the requirement for informed consent was waived by the Ethics Committee. Patient confidentiality was maintained throughout the study, and all data were anonymized prior to analysis.

## 3. Results

### 3.1. Study Population and Baseline Characteristics

A total of 120 patients were included in the analysis, comprising 51 patients in the MA group and 69 patients in the KA group. Baseline demographic, clinical, radiographic, and surgical characteristics are summarized in Table 1.

No statistically significant between-group differences were observed in age, sex distribution, BMI, operated knee side, KL grade, American Society of Anesthesiologists (ASA) physical status, comorbidities, smoking status, preoperative alignment parameters, or patellar resurfacing (all *p* > 0.05). All patients underwent implantation of a cemented prosthesis.

### 3.2. Reliability of Radiographic Measurements

Interobserver and intraobserver reliability was evaluated in a subset of 30 patients. Overall, the measurements demonstrated good-to-excellent reliability. Interobserver ICCs ranged from 0.879 to 0.965, whereas intraobserver ICCs ranged from 0.901 to 0.939.

MAD demonstrated the highest interobserver reliability (ICC = 0.965), followed by radiographic knee extension deficit (ICC = 0.945), HKA angle (ICC = 0.932), and residual varus deformity (ICC = 0.879). For intraobserver reliability, HKA angle demonstrated the highest ICC (0.939), followed by MAD (ICC = 0.938), residual varus deformity (ICC = 0.925), and radiographic knee extension deficit (ICC = 0.901). The ICC estimates and corresponding 95% CIs are presented in Table 2.

### 3.3. Changes in Radiographic Alignment Following TKA

Significant preoperative-to-postoperative changes were observed in the evaluated radiographic alignment and clinical parameters following primary TKA. In the overall cohort of 120 patients, the mean HKA angle increased from 171.19 ± 5.07° preoperatively to 178.83 ± 2.05° postoperatively (*p* < 0.001), representing a mean within-patient change of +7.64 ± 5.38° and an overall shift toward neutral MA.

Mean signed mechanical axis deviation (MAD) changed from −26.95 ± 17.66 mm preoperatively to −3.96 ± 8.14 mm postoperatively, corresponding to a mean change of +22.99 ± 20.40 mm (*p* < 0.001).

Varus deformity decreased from 9.75 ± 4.34° to 2.16 ± 1.47°, with a mean change of −7.59 ± 4.62° (*p* < 0.001). Radiographic knee extension deficit decreased from 8.55 ± 4.48° to 4.00 ± 1.91°, corresponding to a mean change of −4.55 ± 4.88° (*p* < 0.001).

These cohort-level changes are summarized in Table 3. They describe postoperative changes in the evaluated parameters and should not be interpreted as evidence of achievement of an individualized KA target in the KA group.

### 3.4. Functional Outcomes Following TKA

Significant preoperative-to-postoperative improvements were observed in all three functional outcome measures following primary TKA. In the overall cohort of 120 patients, the mean KOOS increased from 41.41 ± 11.67 preoperatively to 81.10 ± 14.20 postoperatively, corresponding to a mean within-patient improvement of 39.69 ± 8.88 points (*p* < 0.001).

Mean WOMAC decreased from 62.03 ± 11.55 preoperatively to 23.32 ± 15.64 postoperatively, with a mean change of −38.70 ± 10.63 points (*p* < 0.001). Mean KSS increased from 44.10 ± 12.16 to 85.04 ± 13.22, corresponding to a mean improvement of 40.94 ± 9.32 points (*p* < 0.001).

**Figure 3:**
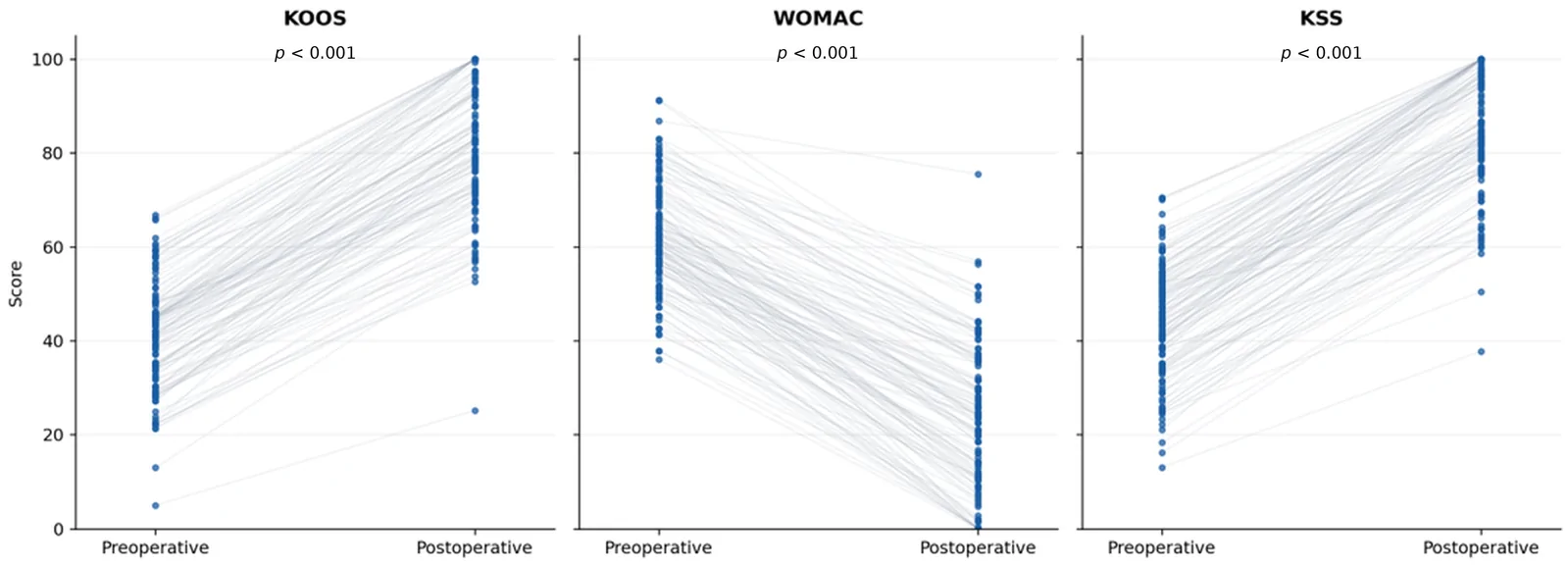
Preoperative and postoperative functional outcomes following primary TKA. Paired plots illustrate individual changes in KOOS, WOMAC, and KSS from the preoperative assessment to the final postoperative follow-up. Each line represents an individual patient. *p*-values were calculated using paired-samples *t*-tests for preoperative-to-postoperative comparisons.

The corresponding paired-samples effect sizes (Cohen’s dz) were 4.47 for KOOS, 3.64 for WOMAC, and 4.39 for KSS. These findings are summarized in Table 4 and illustrated in Figure 3.

### 3.5. Functional and Radiographic Outcomes According to Alignment Strategy

Functional and radiographic outcomes were compared between the MA and KA groups. Both groups demonstrated substantial improvements in functional scores from baseline.

Postoperative KOOS was higher in the KA group than in the MA group (84.04 ± 12.33 vs. 77.12 ± 15.65), corresponding to a mean between-group difference of 6.92 points (KA − MA; 95% CI, 1.67 to 12.18; *p* = 0.010; Cohen’s d = 0.50). However, preoperative KOOS was also higher in the KA group (43.49 ± 11.44 vs. 38.59 ± 11.49; *p* = 0.022), indicating a baseline imbalance. Consequently, the unadjusted postoperative KOOS difference should not be interpreted as an independent effect of alignment strategy without adjustment for baseline scores and relevant covariates.

Postoperative WOMAC scores were numerically lower in the KA group than in the MA group (21.79 ± 16.13 vs. 25.40 ± 14.86), but this difference was not statistically significant (mean difference, −3.62 points; 95% CI, −9.26 to 2.02; *p* = 0.206; Cohen’s d = −0.23). Postoperative KSS scores were also comparable between the KA and MA groups (85.25 ± 13.88 vs. 84.77 ± 12.39), with a mean difference of 0.48 points (95% CI, −4.29 to 5.25; *p* = 0.843; Cohen’s d = 0.04).

The magnitude of functional improvement from baseline did not differ significantly between alignment strategies. The between-group difference in ΔKOOS was 2.02 points (95% CI, −1.27 to 5.30; *p* = 0.227; FDR-adjusted *p* = 0.341). The corresponding difference in ΔWOMAC was −2.89 points (95% CI, −6.64 to 0.87; *p* = 0.130; FDR-adjusted *p* = 0.341), whereas the difference in ΔKSS was 1.34 points (95% CI, −1.99 to 4.68; *p* = 0.427; FDR-adjusted *p* = 0.427). None of the change-from-baseline comparisons were statistically significant after correction for multiple testing.

Regarding postoperative radiographic alignment, the MA group demonstrated a mean HKA angle closer to the neutral mechanical axis than the KA group (179.63 ± 1.23° vs. 178.24 ± 2.32°). The mean between-group difference (KA − MA) was −1.39° (95% CI, −2.04 to −0.74; Cohen’s d = −0.72; unadjusted *p* < 0.001; FDR-adjusted *p* < 0.001).

When postoperative alignment was expressed as the absolute deviation from the neutral mechanical axis (|HKA − 180°|), the MA group demonstrated a significantly smaller deviation than the KA group (0.97 ± 0.83° vs. 2.42 ± 1.62°, respectively). The mean between-group difference (KA − MA) was 1.45° (95% CI, 1.00 to 1.90; Cohen’s d = 1.08; unadjusted *p* < 0.001; FDR-adjusted *p* < 0.001).

Postoperative absolute mechanical axis deviation (|MAD|) was also lower in the MA group than in the KA group (5.19 ± 4.53 vs. 7.86 ± 6.76 mm), corresponding to a mean between-group difference of 2.67 mm (95% CI, 0.62 to 4.71; Cohen’s d = 0.45; unadjusted *p* = 0.011; FDR-adjusted *p* = 0.018).

No statistically significant between-group differences were observed in postoperative residual varus deformity (2.02 ± 1.45° in the MA group vs. 2.27 ± 1.48° in the KA group; FDR-adjusted *p* = 0.440) or radiographic knee extension deficit (3.94 ± 1.91° vs. 4.04 ± 1.92°, respectively; FDR-adjusted *p* = 0.787).

Overall, unadjusted between-group comparisons demonstrated statistically significant differences in postoperative KOOS, HKA, absolute HKA deviation, and absolute MAD, whereas postoperative WOMAC, KSS, and change-from-baseline functional outcomes did not differ significantly between alignment strategies. The postoperative KOOS difference should be interpreted cautiously because of the significant baseline imbalance between groups and should not be considered evidence of an independent effect of alignment strategy. The radiographic findings indicate that MA achieved postoperative alignment closer to the neutral mechanical axis of 180°. However, deviation from neutral MA does not necessarily represent an alignment error in KA, and these measurements should not be interpreted as indicators of accuracy in achieving individualized KA targets.

**Figure 4:**
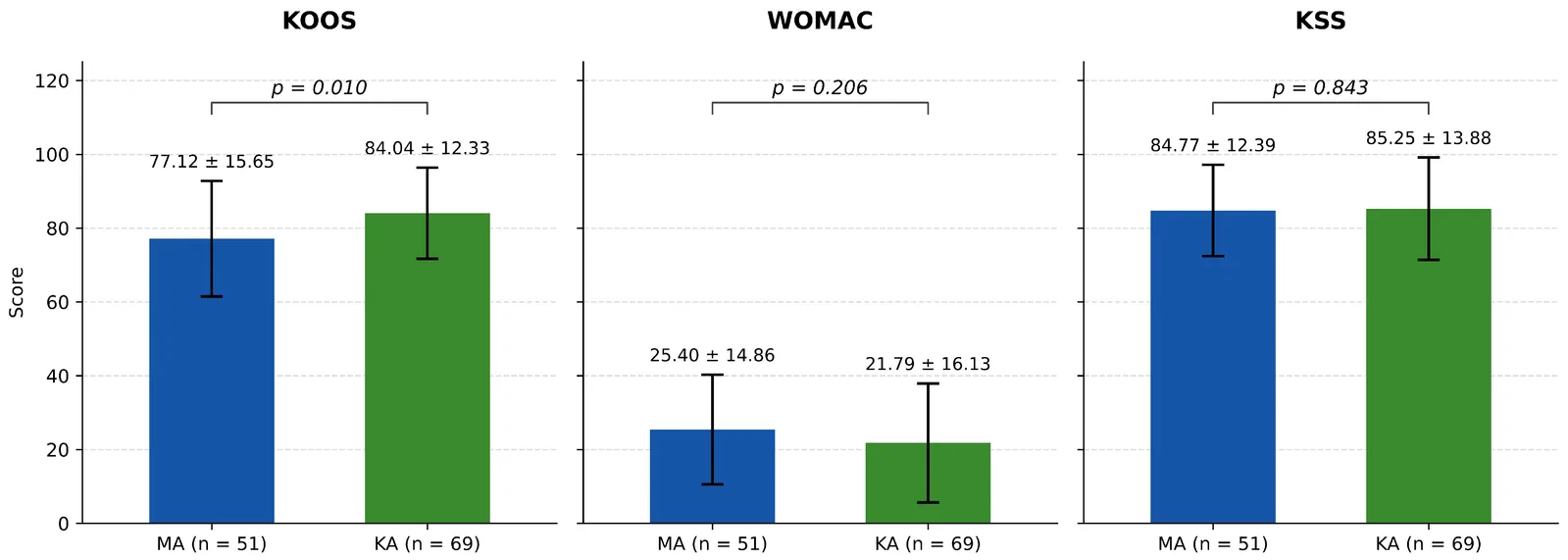
Unadjusted postoperative functional outcomes according to alignment strategy. Bars represent mean postoperative KOOS, WOMAC, and KSS scores in the MA (*n* = 51) and KA (*n* = 69) groups; error bars indicate one SD. *p*-values were calculated using Welch’s independent-samples *t*-tests and represent unadjusted between-group comparisons.

Detailed between-group comparisons are presented in Table 5, and unadjusted postoperative functional outcomes are shown in Figure 4.

### 3.6. Adjusted Analysis of Postoperative Functional Outcomes

To account for measured baseline differences and potential confounding, separate ANCOVA models were fitted for postoperative KOOS, WOMAC, and KSS. Alignment strategy (MA vs. KA) was the main independent variable, with adjustment for the corresponding preoperative functional score, age, sex, BMI, KL grade, and preoperative HKA angle.

In the analysis, KA was associated with a numerically higher postoperative KOOS score compared with MA, but the adjusted difference was not statistically significant (adjusted *β* = 2.42; 95% CI, −0.93 to 5.77; *p* = 0.155). The adjusted difference in postoperative WOMAC was also not statistically significant (adjusted *β* = −2.80; 95% CI, −6.73 to 1.14; *p* = 0.162). Similarly, the adjusted difference in postoperative KSS did not reach statistical significance (adjusted *β* = 1.09; 95% CI, −2.05 to 4.24; *p* = 0.492).

The corresponding preoperative functional score was significantly associated with the postoperative score in each model (all *p* < 0.001). Overall, no statistically significant independent association between alignment strategy and postoperative KOOS, WOMAC, or KSS was observed after adjustment for the included covariates (Table 6).

### 3.7. Postoperative Alignment Relative to the Neutral Mechanical Axis

Postoperative alignment was further characterized according to deviation from the neutral mechanical axis. An HKA angle within ±3° of neutral (180°) was observed in 50 of 51 patients (98.0%) in the MA group and 51 of 69 patients (73.9%) in the KA group (unadjusted *p* < 0.001; FDR-adjusted *p* < 0.001). An absolute mechanical axis deviation (|MAD|) <5 mm was observed in 27 of 51 patients (52.9%) in the MA group and 28 of 69 patients (40.6%) in the KA group (unadjusted *p* = 0.199; FDR-adjusted *p* = 0.199).

HKA deviations >5° from neutral occurred in no patients (0.0%) in the MA group and 4 patients (5.8%) in the KA group. This between-group difference was not statistically significant (unadjusted *p* = 0.136; FDR-adjusted *p* = 0.199).

**Figure 5:**
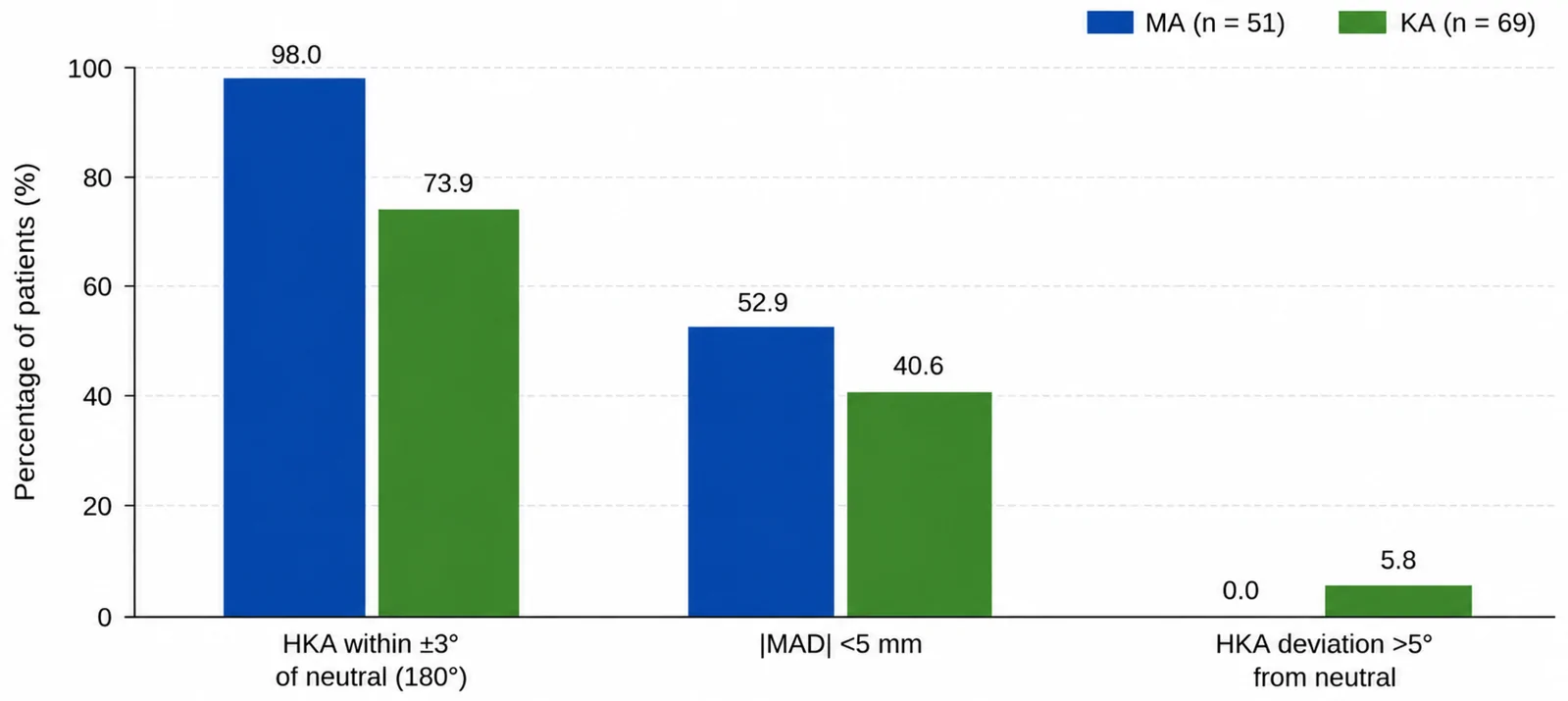
Postoperative alignment relative to the neutral mechanical axis according to alignment strategy. Bars represent the percentage of patients with HKA within ±3° of neutral (180°), |MAD| < 5 mm, and HKA deviations > 5° from neutral in the MA (*n* = 51) and KA (*n* = 69) groups. These categories describe alignment relative to the neutral mechanical axis and do not represent achievement or failure of the individualized KA target.

These analyses characterize postoperative alignment relative to the neutral mechanical axis only. In the KA group, deviation from an HKA angle of 180° should not be interpreted as a technical error or failure to achieve the intended alignment strategy, because deviation from the individualized KA target was not quantitatively assessed in the present study. Postoperative alignment categories are summarized in Table 7 and illustrated in Figure 5.

### 3.8. Relationship Between Postoperative Mechanical-Axis Alignment and Functional Outcomes

To investigate the relationship between postoperative mechanical-axis alignment and functional outcomes, correlation analyses were performed between the measured postoperative radiographic parameters and postoperative KOOS, WOMAC, and KSS scores.

Postoperative HKA showed negligible to weak correlations with KOOS (*r* = −0.009; FDR-adjusted *p* = 0.979), WOMAC (*r* = 0.173; FDR-adjusted *p* = 0.706), and KSS (*r* = 0.105; FDR-adjusted *p* = 0.924). None of these associations reached statistical significance after FDR correction. Similarly, signed postoperative MAD was not significantly associated with KOOS (*r* = −0.052; FDR-adjusted *p* = 0.979), WOMAC (*r* = 0.139; FDR-adjusted *p* = 0.785), or KSS (*r* = 0.039; FDR-adjusted *p* = 0.979).

Residual varus deformity was not significantly associated with any of the three functional outcomes after FDR correction. Radiographic knee extension deficit also showed negligible correlations with KOOS (*r* = −0.026; FDR-adjusted *p* = 0.979), WOMAC (*r* = 0.044; FDR-adjusted *p* = 0.979), and KSS (*r* = 0.012; FDR-adjusted *p* = 0.979).

Overall, none of the 12 correlations between postoperative radiographic parameters and functional outcomes reached statistical significance after FDR correction. Detailed correlation coefficients and corresponding unadjusted and FDR-adjusted *p*-values are presented in Table 8.

Additional exploratory analyses examined whether the magnitude of postoperative MAD, irrespective of direction, was associated with postoperative functional outcomes or changes from baseline. Absolute HKA deviation was calculated as |HKA − 180°|, and absolute MAD was defined as |MAD|.

In the overall cohort, absolute HKA deviation was not significantly correlated with postoperative KOOS (*r* = 0.138; FDR-adjusted *p* = 0.402), WOMAC (*r* = −0.089; FDR-adjusted *p* = 0.458), or KSS (*r* = −0.142; FDR-adjusted *p* = 0.402). Similarly, absolute MAD showed no statistically significant associations with postoperative KOOS, WOMAC, or KSS.

Neither absolute HKA deviation nor absolute MAD was significantly associated with changes from baseline in KOOS, WOMAC, or KSS after FDR correction (all FDR-adjusted *p* = 0.582). Correlations stratified by alignment strategy were generally negligible or weak in both the MA and KA groups.

Overall, no statistically significant associations were identified between the magnitude of postoperative MAD and postoperative functional status or change-from-baseline scores in these exploratory analyses. However, these findings should not be interpreted as evidence of an absence of association, particularly given the sample size and exploratory nature of the subgroup analyses. Detailed results are presented in Table 9.

### 3.9. Follow-Up, Postoperative Complications, and Reoperations

The mean postoperative follow-up was 25.2 ± 8.1 months for the overall cohort (range, 12–43 months) and was similar in the MA and KA groups (25.6 ± 8.3 and 24.9 ± 8.0 months, respectively).

During the available follow-up period, postoperative complications and reoperations were retrospectively identified from the medical records. PJI occurred in 2 patients (1.7%), including 1 patient in each alignment group. Reoperation for any reason was recorded in 3 patients (2.5%), including 1 patient in the MA group and 2 patients in the KA group. Aseptic loosening was identified in 1 patient (0.8%), in the KA group, and revision TKA was performed in 2 patients (1.7%), including 1 patient in each group. Follow-up duration and observed adverse events are summarized in Table 10.

Given the small number of events and the limited duration of follow-up, complications and reoperations were analyzed descriptively, without inferential between-group comparisons. These findings should not be interpreted as evidence of equivalence in complication rates or comparative long-term implant safety, survivorship, or revision risk between MA and KA.

## 4. Discussion

The present study compared short-term functional outcomes and postoperative mechanical-axis alignment between MA and KA following primary TKA and investigated the relationship between measured postoperative alignment parameters and functional outcomes. The principal findings were fourfold. First, both alignment strategies were associated with substantial improvement in functional outcomes following TKA. Second, MA achieved postoperative alignment closer to the neutral mechanical axis, whereas KA was associated with a statistically significantly higher postoperative KOOS score in the unadjusted comparison, while postoperative KSS and WOMAC did not differ significantly between groups. However, the KOOS difference was not statistically significant after adjustment for baseline covariates, and no statistically significant differences were observed in the magnitude of functional improvement from baseline. Third, 98.0% of patients in the MA group and 73.9% in the KA group had postoperative HKA values within ±3° of neutral, with deviations exceeding this threshold being more frequent in the KA group. Finally, associations between postoperative radiographic alignment parameters and functional outcomes were generally weak and did not remain statistically significant after correction for multiple comparisons, suggesting that mechanical-axis alignment relative to neutral alone may not adequately account for short-term functional recovery following TKA.

Importantly, the radiographic findings in the KA group should be interpreted only as measures of postoperative alignment relative to the neutral mechanical axis rather than as indicators of successful restoration of patient-specific anatomy. Because mPTA, LDFA, JLO, and CPAK phenotype were not systematically measured or recorded in the original dataset, and achievement of the individualized KA target was not quantitatively assessed, the present study could not determine whether native coronal alignment or the intended patient-specific alignment target had been restored. Consequently, the weak associations observed between postoperative mechanical-axis parameters and functional outcomes should not be interpreted as evidence for or against the functional benefits of restoring patient-specific knee anatomy.

### 4.1. Radiographic Alignment Following TKA

Significant preoperative-to-postoperative improvements were observed in all measured radiographic parameters, including HKA angle, MAD, residual varus deformity, and radiographic knee extension deficit, reflecting substantial correction of preoperative lower-limb deformity following primary TKA. These findings are consistent with previous investigations reporting substantial postoperative correction of lower-limb deformity following TKA [32,33,34].

MA has historically been considered the reference alignment strategy in TKA because restoration of a neutral mechanical axis has been proposed to promote balanced load distribution, reduce polyethylene wear, and support long-term implant survivorship [12,14,17]. Consistent with this objective, MA resulted in postoperative HKA values closer to the neutral mechanical axis than KA in the present cohort (absolute HKA deviation: 0.97 ± 0.83° vs. 2.42 ± 1.62°; unadjusted and FDR-adjusted *p* < 0.001). Similarly, |MAD| was significantly lower in the MA group, and this difference remained statistically significant after FDR correction (5.19 ± 4.53 vs. 7.86 ± 6.76 mm; unadjusted *p* = 0.011; FDR-adjusted *p* = 0.018). These findings indicate that the two alignment strategies were associated with distinct postoperative mechanical-axis profiles, particularly with respect to HKA and absolute MAD.

Importantly, greater deviation from the neutral mechanical axis in the KA group should not be interpreted as inferior radiographic correction or technical failure. KA intentionally permits individualized postoperative alignment rather than systematically targeting an HKA angle of 180°. Because achievement of the individualized KA target was not quantitatively assessed, these findings describe deviation from neutral alignment rather than the accuracy of patient-specific alignment restoration.

### 4.2. Functional Recovery After TKA

The present study demonstrated substantial improvements in all functional outcome measures following primary TKA, with marked changes in KOOS, WOMAC, and KSS from baseline to postoperative assessment. The large within-patient effect sizes observed across these measures further indicate substantial preoperative-to-postoperative functional improvement. These findings are consistent with previous randomized trials and systematic reviews reporting marked improvements in pain, functional capacity, and quality of life following primary TKA [26,27,35,36].

When alignment strategies were compared, patients treated with KA had a statistically significantly higher unadjusted postoperative KOOS score than those treated with MA, whereas postoperative KSS and WOMAC scores did not differ significantly between groups. Similar modest differences in selected patient-reported outcomes between KA and MA have been reported in previous studies [25,37,38]. However, the unadjusted postoperative KOOS difference should be interpreted in the context of baseline functional differences and the absence of a statistically significant adjusted association. Statistical significance should therefore be distinguished from clinical significance. Published estimates of the minimal clinically important difference (MCID) vary according to the population, follow-up period, outcome definition, and method used to derive the threshold [39,40]. Accordingly, the observed differences should be interpreted cautiously and, without a prespecified, population-appropriate MCID threshold, should not be considered evidence of clinically meaningful superiority of KA based on statistical significance alone.

This interpretation is further supported by the change-from-baseline analyses. Although both groups experienced substantial functional improvement, between-group differences in change from baseline were not statistically significant for any of the three functional outcomes. Thus, both strategies were associated with substantial functional improvement, but the magnitude of improvement from baseline did not differ significantly between MA and KA. Nevertheless, the absence of statistically significant differences in change scores should not be interpreted as evidence of equivalence between the two strategies.

After adjustment for the corresponding baseline functional score, age, sex, BMI, KL grade, and preoperative HKA angle, no statistically significant independent association was observed between alignment strategy and postoperative KOOS (adjusted *β* = 2.42; 95% CI, −0.93 to 5.77; *p* = 0.155), WOMAC (adjusted *β* = −2.80; 95% CI, −6.73 to 1.14; *p* = 0.162), or KSS (adjusted *β* = 1.09; 95% CI, −2.05 to 4.24; *p* = 0.492). Although the adjusted point estimates were numerically more favorable in the KA group, the CIs included the null value, and the findings did not establish an independent functional advantage of KA over MA. Moreover, the retrospective, non-randomized design remains susceptible to residual confounding and confounding by indication, precluding causal conclusions regarding the comparative effectiveness of the two alignment strategies.

### 4.3. Alignment Strategy and Postoperative Mechanical-Axis Alignment

MA achieved postoperative alignment closer to the neutral mechanical axis than KA, with significantly smaller absolute HKA deviation and |MAD| (5.19 ± 4.53 mm vs. 7.86 ± 6.76 mm; FDR-adjusted *p* = 0.018). An HKA angle within ±3° of neutral was observed in 98.0% of patients in the MA group and 73.9% in the KA group, while deviations exceeding 5° were uncommon (0.0% and 5.8%, respectively). These findings are consistent with previous studies reporting greater variability in postoperative mechanical-axis alignment with individualized alignment strategies [41,42,43].

Nevertheless, greater deviation from neutral in the KA group should not be interpreted as evidence of technical failure, because achievement of the intended patient-specific alignment target was not quantitatively assessed. Furthermore, mPTA, LDFA, JLO, and CPAK phenotype were not systematically measured or recorded in the original dataset, limiting the evaluation of restoration of constitutional alignment. Future studies incorporating patient-specific alignment targets and detailed postoperative coronal measurements are needed to assess the accuracy of individualized alignment strategies.

### 4.4. Relationship Between Radiographic Alignment and Functional Outcomes

Another important finding of the present study was the limited relationship between the measured postoperative radiographic parameters and functional outcomes. Postoperative HKA and signed MAD showed generally weak or negligible correlations with functional outcomes, while analyses based on absolute deviation from the neutral mechanical axis (|HKA − 180°| and |MAD|) demonstrated a similar pattern. The directions of these associations were not consistent across functional outcome measures. Radiographic knee extension deficit also showed negligible associations with all three functional outcomes, whereas residual varus deformity showed no statistically significant associations with any functional outcome. Overall, none of the correlations between the measured postoperative radiographic parameters and functional outcomes remained statistically significant after Benjamini–Hochberg FDR correction, suggesting that these parameters showed limited associations with short-term functional outcomes in the present cohort.

Importantly, these correlations should be interpreted as associations between the measured postoperative radiographic parameters and functional outcomes rather than as evidence that achievement of a particular alignment strategy determines clinical recovery. Because the individualized KA target could not be quantitatively evaluated, these analyses cannot establish whether closer restoration of patient-specific alignment was associated with better functional outcomes or support causal conclusions regarding alignment and postoperative recovery.

These findings are consistent with the concept that functional recovery after TKA is multifactorial and may depend on factors beyond postoperative mechanical-axis alignment, including soft-tissue balance, muscle strength, rehabilitation, psychological factors, pain perception, and patient expectations [44,45,46,47]. Accordingly, the weak or negligible associations observed in the present study suggest that postoperative mechanical-axis alignment alone may not adequately explain variation in short-term functional outcomes. This finding should not be interpreted as indicating that alignment is clinically unimportant, but rather that the relationship between radiographic alignment and postoperative function is likely more complex than can be captured by HKA, MAD, residual varus deformity, and radiographic knee extension deficit alone.

### 4.5. Clinical Implications

Both MA and KA were associated with substantial short-term functional improvement. Although postoperative complications and reoperations were uncommon during the available follow-up, the small number of events and limited observation period preclude conclusions regarding comparative safety or implant survivorship.

The higher unadjusted postoperative KOOS score observed with KA was not statistically significant after adjustment for baseline covariates, and neither adjusted WOMAC and KSS scores nor changes from baseline differed significantly between groups. Moreover, the clinical relevance of the unadjusted KOOS difference remains uncertain, as published MCID thresholds vary across populations and assessment methods and cannot necessarily be applied to between-group comparisons. Therefore, the present findings do not establish a clinically meaningful functional advantage of either alignment strategy.

The weak associations between postoperative mechanical-axis parameters and functional outcomes indicate that radiographic alignment should be interpreted alongside other determinants of recovery rather than as an isolated measure of clinical success. Accordingly, the present observational findings do not support preferential use of MA or KA. Selection of an alignment strategy should consider individual patient anatomy, clinical characteristics, surgical objectives, and surgeon experience, while recognizing that longer-term comparative studies are needed to establish their relative effectiveness and safety.

### 4.6. Study Limitations

Several limitations should be considered when interpreting the findings of the present study. First, this was a single-center study, which may limit the generalizability of the findings to other institutions, surgical teams, and patient populations. Multicenter studies involving different surgeons and healthcare settings are needed to assess the reproducibility of these findings.

Second, the sample size was determined by the number of eligible patients available in the retrospective cohort and was not based on an a priori sample-size or power calculation. Consequently, the available sample size may have limited the ability to detect small between-group differences, particularly for secondary outcomes, subgroup analyses, and uncommon complications or revision events. Larger prospective studies with predefined sample-size calculations would provide more precise estimates of between-group differences.

Third, the available follow-up primarily reflects short-term clinical and radiographic outcomes and is insufficient to evaluate long-term arthroplasty outcomes. Although the minimum 12-month follow-up permitted assessment of postoperative alignment and functional recovery, it does not allow meaningful evaluation of implant survivorship, polyethylene wear, aseptic loosening, or revision risk. Moreover, the small number of observed complications and reoperations precluded meaningful between-group comparisons. Longer-term studies, ideally with at least 5 years of follow-up and systematic assessment of complications, revision events, and implant survivorship, are required.

Fourth, the retrospective observational design and non-random allocation of alignment strategy introduce the potential for selection bias, confounding by indication, and residual confounding. Furthermore, the exclusion of patients with incomplete clinical or radiographic data or insufficient follow-up may have introduced additional selection bias, as the analyzed cohort may not fully represent all patients undergoing TKA during the study period. The choice between MA and KA was made as part of routine clinical decision-making and may have been influenced by patient anatomy, degree of deformity, anticipated soft-tissue balance, and surgeon preference. Although several baseline characteristics were comparable between groups, differences in preoperative functional scores were observed. Adjusted analyses accounted for selected baseline covariates, but these adjustments cannot fully eliminate residual confounding or account for unmeasured factors. Variables such as soft-tissue balancing, patient activity level, rehabilitation adherence, and surgeon-related factors may also have influenced postoperative outcomes. Although all three participating surgeons performed both MA and KA procedures, the number of interventions performed by each surgeon in the two groups was not quantified, preventing assessment or adjustment for surgeon-specific effects. Furthermore, differences in prior experience with the two alignment techniques may have influenced surgical outcomes. Although both strategies were used throughout the study period, potential temporal changes in surgical practice could not be fully accounted for. Accordingly, the observed associations should not be interpreted as evidence of the causal superiority of either alignment strategy.

Fifth, functional assessment was based on KOOS, WOMAC, and KSS, which provide clinically relevant measures of pain and function but do not fully characterize dynamic knee biomechanics. Objective assessments such as gait analysis, dynamic fluoroscopy, muscle strength testing, wearable sensor technology, or instrumented laxity measurements were not available and could provide complementary information in future studies.

Sixth, an important limitation concerns the radiographic characterization of KA. Although HKA, MAD, residual varus deformity, and radiographic knee extension deficit characterize aspects of postoperative coronal alignment and knee extension, they do not establish whether the intended patient-specific KA target was achieved. Preoperative and postoperative mPTA, LDFA, JLO, and CPAK phenotype were not systematically measured or recorded in the original dataset and therefore could not be analyzed. Moreover, deviation from the individualized KA target was not quantitatively assessed. Although the participating surgeons reported following common technical principles, patient-specific planned component orientations and adherence to the intended alignment targets were not systematically documented. Therefore, potential variability in the implementation of the KA technique across individual procedures and surgeons could not be independently assessed. Consequently, neither restoration of constitutional coronal anatomy nor accuracy in achieving the intended KA target could be established. This limitation also prevents the alignment–function analyses from determining whether closer achievement of patient-specific alignment targets was associated with better functional outcomes.

Finally, the study was not designed or powered to determine whether specific patient subgroups, such as patients with severe constitutional varus deformity, obesity, or different degrees of preoperative functional impairment, derive greater benefit from one alignment strategy than the other. Future adequately powered studies should investigate potential effect modification across clinically relevant patient subgroups.

### 4.7. Future Directions

Future research should prioritize prospective, adequately powered multicenter studies with longer follow-up to determine whether clinically meaningful functional differences emerge between MA and KA over time and whether either strategy is associated with differences in complications, implant survivorship, or revision risk. Randomized designs, where feasible, would further reduce the potential influence of selection bias and confounding inherent to retrospective comparisons.

Future studies should also incorporate comprehensive patient-specific coronal alignment assessment, including mPTA, LDFA, JLO, and CPAK phenotype, together with quantitative comparison between the planned alignment target and the postoperative alignment achieved. Such data would allow investigators to distinguish deviation from the neutral mechanical axis from failure to achieve an individualized alignment target and to determine whether accurate restoration of patient-specific anatomy is associated with improved functional outcomes. Evaluation of constitutional limb alignment, deformity severity, ligament balance, bone morphology, BMI, activity level, and patient expectations may further help identify subgroups that derive greater benefit from specific alignment strategies.

Finally, combining validated patient-reported outcomes with objective functional and biomechanical measures, including gait analysis, muscle strength testing, wearable motion sensors, dynamic imaging, and in vivo knee kinematics, may provide a more comprehensive understanding of the relationship between alignment and postoperative recovery. Advanced surgical technologies, including robotic assistance, computer navigation, three-dimensional planning, and data-driven surgical planning, may facilitate more precise implementation and evaluation of individualized alignment strategies; however, their clinical value should be assessed in appropriately designed comparative studies rather than inferred from improvements in technical precision alone.

## 5. Conclusions

Both MA and KA were associated with substantial short-term functional improvement following primary TKA. MA achieved postoperative alignment closer to the neutral mechanical axis, whereas KA was associated with a statistically significantly higher postoperative KOOS score in the unadjusted comparison, with no significant differences in postoperative WOMAC or KSS scores. However, the KOOS difference was not statistically significant after adjustment for baseline covariates, and improvement from baseline did not differ significantly between alignment strategies. Accordingly, the present findings do not demonstrate a statistically significant adjusted functional advantage or establish clinically meaningful short-term superiority of either strategy.

Associations between the measured postoperative mechanical-axis parameters and functional outcomes were generally weak or negligible and did not remain statistically significant after correction for multiple comparisons, suggesting that mechanical-axis alignment alone may not adequately explain variation in short-term functional recovery. Because patient-specific coronal alignment parameters and achievement of the individualized KA target were not quantitatively assessed, these findings cannot determine whether restoration of patient-specific anatomy is associated with improved functional outcomes. Longer-term, adequately powered prospective studies incorporating comprehensive patient-specific alignment assessment are required to determine whether differences between MA and KA persist over time and influence implant-related outcomes.

## Figures and Tables

**Table 1:** Baseline demographic, clinical, radiographic, and surgical characteristics according to alignment strategy.

Variable	Overall (*n* = 120)	MA (*n* = 51)	KA (*n* = 69)	*p*-Value
**Demographic characteristics**				
Age (years), mean ± SD	67.83 ± 6.69	67.86 ± 6.51	67.81 ± 6.87	0.967
Female sex, *n* (%)	72 (60.00)	30 (58.82)	42 (60.87)	0.821
BMI (kg/m^2^), mean ± SD	30.84 ± 4.23	31.13 ± 4.17	30.62 ± 4.29	0.518
**Clinical characteristics**				
Right knee, *n* (%)	66 (55.00)	28 (54.90)	38 (55.07)	0.985
KL grade III, *n* (%)	34 (28.33)	15 (29.41)	19 (27.54)	0.822 *
KL grade IV, *n* (%)	86 (71.67)	36 (70.59)	50 (72.46)	
ASA physical status I, *n* (%)	18 (15.00)	7 (13.73)	11 (15.94)	0.933 †
ASA physical status II, *n* (%)	71 (59.17)	31 (60.78)	40 (57.97)	
ASA physical status III, *n* (%)	31 (25.83)	13 (25.49)	18 (26.09)	
Diabetes mellitus, *n* (%)	27 (22.50)	12 (23.53)	15 (21.74)	0.816
Hypertension, *n* (%)	81 (67.50)	35 (68.63)	46 (66.67)	0.821
Current smokers, *n* (%)	18 (15.00)	8 (15.69)	10 (14.49)	0.856
**Preoperative radiographic characteristics**				
HKA angle (°), mean ± SD	171.19 ± 5.07	171.25 ± 4.63	171.15 ± 5.39	0.919
MAD (mm), mean ± SD	−26.95 ± 17.66	−28.88 ± 17.54	−25.52 ± 17.74	0.305
Varus deformity (°), mean ± SD	9.75 ± 4.34	10.43 ± 3.91	9.25 ± 4.59	0.130
Radiographic knee extension deficit (°), mean ± SD	8.55 ± 4.48	8.32 ± 4.59	8.73 ± 4.42	0.619
**Surgical characteristics**				
Patellar resurfacing, *n* (%)	39 (32.50)	17 (33.33)	22 (31.88)	0.867
Cemented prosthesis, *n* (%)	120 (100.00)	51 (100.00)	69 (100.00)	N/A

Note: Data are presented as mean ± SD or number (%). Continuous variables were compared using Welch’s independent-samples *t*-tests. Categorical variables were compared using Pearson’s chi-square test. * The *p*-value refers to the overall comparison of KL grade distribution between groups. † The *p*-value refers to the overall comparison of ASA physical status distribution between groups. A *p*-value was not calculated for cemented prosthesis because all patients belonged to the same category. Statistical significance was defined as *p* < 0.05.

**Table 2:** Intraobserver and interobserver reliability of radiographic measurements.

Measurement Parameter	Interobserver ICC (95% CI)	Intraobserver ICC (95% CI)
HKA angle	0.932 (0.847–0.967)	0.939 (0.861–0.969)
MAD	0.965 (0.899–0.983)	0.938 (0.810–0.972)
Residual varus deformity	0.879 (0.719–0.953)	0.925 (0.855–0.959)
Radiographic knee extension deficit	0.945 (0.902–0.967)	0.901 (0.822–0.945)

Note: Interobserver and intraobserver reliability were assessed in a subset of 30 patients using a two-way absolute-agreement, single-measurement ICC model ICC(A,1). Interobserver reliability was evaluated using independent measurements performed by two observers, whereas intraobserver reliability was evaluated using repeated measurements performed by the first observer after a 4-week interval. The 95% CIs were estimated using patient-level bootstrap resampling (5000 iterations).

**Table 3:** Preoperative-to-postoperative changes in radiographic alignment and radiographic knee extension deficit following primary TKA.

Parameter	Preoperative	Postoperative	Mean Change (Post − Pre)	*p*-Value
HKA angle (°)	171.19 ± 5.07	178.83 ± 2.05	+7.64 ± 5.38	<0.001
Signed MAD (mm)	−26.95 ± 17.66	−3.96 ± 8.14	+22.99 ± 20.40	<0.001
Varus deformity (°)	9.75 ± 4.34	2.16 ± 1.47	−7.59 ± 4.62	<0.001
Radiographic knee extension deficit (°)	8.55 ± 4.48	4.00 ± 1.91	−4.55 ± 4.88	<0.001

Note: Data are presented as mean ± SD for the overall cohort (*n* = 120). Mean changes were calculated as postoperative minus preoperative values for each patient. Statistical comparisons were performed using paired-samples *t*-tests. Negative MAD values indicate medial deviation (varus), whereas positive values indicate lateral deviation (valgus). Statistical significance was defined as *p* < 0.05.

**Table 4:** Preoperative-to-postoperative changes in functional outcome measures following primary TKA.

Outcome Measure	Preoperative	Postoperative	Mean Change (Post − Pre)	*p*-Value	Cohen’s d_z_
KOOS	41.41 ± 11.67	81.10 ± 14.20	+39.69 ± 8.88	<0.001	4.47
WOMAC	62.03 ± 11.55	23.32 ± 15.64	−38.70 ± 10.63	<0.001	3.64
KSS	44.10 ± 12.16	85.04 ± 13.22	+40.94 ± 9.32	<0.001	4.39

Note: Data are presented as mean ± SD for the overall cohort (*n* = 120). Mean changes were calculated as postoperative minus preoperative values. Positive changes in KOOS and KSS and negative changes in WOMAC indicate functional improvement. Preoperative-to-postoperative comparisons were performed using paired-samples *t*-tests. Cohen’s d was calculated as the mean within-patient change divided by the SD of the paired changes; absolute effect-size values are presented. Statistical significance was defined as *p* < 0.05.

**Table 5:** Comparison of functional and radiographic outcomes between the MA and KA groups.

Outcome	MA (*n* = 51)	KA (*n* = 69)	Mean Difference (KA − MA) 95% CI	Cohen’s d	*p*-Value	FDR-Adjusted *p*
**KOOS**						
Preoperative	38.59 ± 11.49	43.49 ± 11.44	4.91 (0.71 to 9.11)	0.43	0.022	—
Postoperative	77.12 ± 15.65	84.04 ± 12.33	6.92 (1.67 to 12.18)	0.50	0.010	—
Δ (post − pre)	38.53 ± 9.31	40.55 ± 8.51	2.02 (−1.27 to 5.30)	0.23	0.227	0.341
**WOMAC**						
Preoperative	62.45 ± 11.67	61.72 ± 11.53	−0.73 (−4.98 to 3.52)	−0.06	0.734	—
Postoperative	25.40 ± 14.86	21.79 ± 16.13	−3.62 (−9.26 to 2.02)	−0.23	0.206	—
Δ (post − pre)	−37.04 ± 9.31	−39.93 ± 11.42	−2.89 (−6.64 to 0.87)	−0.27	0.130	0.341
**KSS**						
Preoperative	44.60 ± 10.81	43.73 ± 13.13	−0.86 (−5.20 to 3.47)	−0.07	0.694	—
Postoperative	84.77 ± 12.39	85.25 ± 13.88	0.48 (−4.29 to 5.25)	0.04	0.843	—
Δ (post − pre)	40.17 ± 8.50	41.51 ± 9.90	1.34 (−1.99 to 4.68)	0.14	0.427	0.427
**Postoperative alignment**						
HKA (°)	179.63 ± 1.23	178.24 ± 2.32	−1.39 (−2.04 to −0.74)	−0.72	<0.001	<0.001
Absolute HKA deviation (°)	0.97 ± 0.83	2.42 ± 1.62	1.45 (1.00 to 1.90)	1.08	<0.001	<0.001
Absolute MAD (mm)	5.19 ± 4.53	7.86 ± 6.76	2.67 (0.62 to 4.71)	0.45	0.011	0.018
Residual varus (°)	2.02 ± 1.45	2.27 ± 1.48	0.25 (−0.28 to 0.79)	0.17	0.352	0.440
Radiographic knee extension deficit (°)	3.94 ± 1.91	4.04 ± 1.92	0.10 (−0.61 to 0.80)	0.05	0.787	0.787

Note: Data are presented as mean ± SD. Mean differences are expressed as KA − MA. Δ represents postoperative minus preoperative values; positive changes in KOOS and KSS and negative changes in WOMAC indicate improvement. Absolute HKA deviation was calculated for each patient as |postoperative HKA − 180°|, irrespective of varus or valgus direction. Cohen’s d represents the standardized between-group difference calculated using the pooled SD. Between-group comparisons were calculated using Welch’s independent-samples *t*-tests. The Benjamini–Hochberg FDR procedure was applied to the three functional change-from-baseline comparisons and the five postoperative radiographic comparisons. An em dash indicates that FDR correction was not applied.

**Table 6:** Adjusted association between alignment strategy and postoperative functional outcomes.

Postoperative Outcome	Adjusted *β* for KA vs. MA	95% CI	*p*-Value
KOOS	2.42	−0.93 to 5.77	0.155
WOMAC	−2.80	−6.73 to 1.14	0.162
KSS	1.09	−2.05 to 4.24	0.492

Note: Data are presented as adjusted regression coefficients (*β*) with 95% CIs. MA was the reference category. Separate ANCOVA models were constructed for postoperative KOOS, WOMAC, and KSS, with adjustment for the corresponding preoperative functional score, age, sex, BMI, KL grade, and preoperative HKA angle. Positive β values for KOOS and KSS indicate higher postoperative scores in the KA group, whereas a negative β value for WOMAC indicates a lower postoperative score in the KA group. Statistical significance was defined as *p* < 0.05.

**Table 7:** Postoperative alignment relative to the neutral mechanical axis according to alignment strategy.

Radiographic Outcome	MA Group (*n* = 51)	KA Group (*n* = 69)	Unadjusted *p*	FDR-Adjusted *p*
HKA within ±3° of neutral (180°), *n* (%)	50 (98.0)	51 (73.9)	<0.001	<0.001
|MAD| < 5 mm, *n* (%)	27 (52.9)	28 (40.6)	0.199	0.199
HKA deviation > 5° from neutral, *n* (%)	0 (0.0)	4 (5.8)	0.136	0.199

Note: Data are presented as number (%). Neutral MA was defined as an HKA angle of 180°. HKA deviations were calculated relative to the neutral mechanical axis and should not be interpreted as deviations from the individualized KA target or as technical errors in achieving the intended KA strategy. Between-group comparisons were performed using Fisher’s exact test. The Benjamini–Hochberg FDR procedure was applied across the three comparisons. Statistical significance after correction was defined as an FDR-adjusted *p* < 0.05.

**Table 8:** Correlations between postoperative radiographic parameters and functional outcomes.

Radiographic Parameter	Functional Outcome	*r*	Unadjusted *p*	FDR-Adjusted *p*
Postoperative HKA angle	KOOS	−0.009	0.923	0.979
	WOMAC	0.173	0.059	0.706
	KSS	0.105	0.253	0.924
Postoperative MAD (signed)	KOOS	−0.052	0.570	0.979
	WOMAC	0.139	0.131	0.785
	KSS	0.039	0.676	0.979
Residual varus deformity	KOOS	0.094	0.308	0.924
	WOMAC	−0.003	0.973	0.979
	KSS	−0.002	0.979	0.979
Radiographic knee extension deficit	KOOS	−0.026	0.781	0.979
	WOMAC	0.044	0.636	0.979
	KSS	0.012	0.897	0.979

Note: Correlations were assessed using Pearson’s correlation coefficient (*r*). MAD was analyzed as a signed measure of mechanical-axis displacement in this analysis. Positive coefficients indicate direct associations and negative coefficients indicate inverse associations. The Benjamini–Hochberg FDR procedure was applied across the 12 correlation tests (four radiographic parameters × three functional outcomes). Both unadjusted and FDR-adjusted *p*-values are reported. Statistical significance after correction was defined as an FDR-adjusted *p* < 0.05.

**Table 9:** Associations of absolute postoperative HKA and MAD deviations with functional outcomes and change-from-baseline scores.

Alignment Parameter	Outcome	Overall *r* (95% CI)	FDR-Adjusted *p*	MA *r*	KA *r*
|HKA − 180°|	Postop KOOS	0.138 (−0.043 to 0.309)	0.402	0.012	0.038
	Postop WOMAC	−0.089 (−0.264 to 0.092)	0.458	0.168	−0.113
	Postop KSS	−0.142 (−0.313 to 0.038)	0.402	−0.210	−0.164
|MAD|	Postop KOOS	0.068 (−0.112 to 0.245)	0.458	−0.058	0.064
	Postop WOMAC	−0.075 (−0.251 to 0.106)	0.458	0.120	−0.130
	Postop KSS	−0.097 (−0.271 to 0.084)	0.458	−0.019	−0.142
|HKA − 180°|	ΔKOOS	0.056 (−0.124 to 0.233)	0.582	−0.067	0.032
	ΔWOMAC	−0.095 (−0.270 to 0.086)	0.582	0.168	−0.100
	ΔKSS	0.064 (−0.116 to 0.241)	0.582	−0.131	0.090
|MAD|	ΔKOOS	−0.051 (−0.228 to 0.130)	0.582	−0.125	−0.056
	ΔWOMAC	−0.076 (−0.252 to 0.104)	0.582	0.179	−0.140
	ΔKSS	0.057 (−0.124 to 0.234)	0.582	0.063	0.034

Note: Absolute HKA deviation was calculated as |postoperative HKA − 180°| and absolute MAD as |postoperative MAD|. Higher absolute values indicate greater deviation from the neutral mechanical axis. Δ was calculated as the postoperative value minus the corresponding preoperative value; therefore, positive Δ values for KOOS and KSS and negative Δ values for WOMAC indicate functional improvement. Correlations were assessed using Pearson’s correlation coefficient (*r*). The 95% CIs for the overall correlation coefficients were calculated using Fisher’s z transformation and were not adjusted for multiple comparisons. The Benjamini–Hochberg FDR procedure was applied separately to two families of six tests: associations with postoperative functional scores and associations with change-from-baseline scores. Analyses stratified by alignment strategy were considered exploratory.

**Table 10:** Follow-up duration and postoperative complications according to alignment strategy.

Outcome	Overall (*n* = 120)	MA (*n* = 51)	KA (*n* = 69)
Follow-up, months, mean ± SD	25.2 ± 8.1	25.6 ± 8.3	24.9 ± 8.0
PJI, *n* (%)	2 (1.7)	1 (2.0)	1 (1.4)
Reoperation for any reason, *n* (%)	3 (2.5)	1 (2.0)	2 (2.9)
Aseptic loosening, *n* (%)	1 (0.8)	0 (0.0)	1 (1.4)
Revision TKA, *n* (%)	2 (1.7)	1 (2.0)	1 (1.4)

Note: Data are presented as mean ± SD or number (%). Postoperative complications and reoperations were analyzed descriptively because of the small number of events and limited duration of follow-up. No inferential comparisons of complication rates between alignment strategies were performed.

## Data Availability

The data presented in this study are available from the corresponding author upon reasonable request. The data are not publicly available due to privacy and ethical restrictions related to patient confidentiality.

## Supplementary Materials

The following supporting information can be downloaded at https://www.mdpi.com/article/10.3390/diagnostics16213446/s1, Supplementary Table S1. Domain-specific KOOS scores before and after total knee arthroplasty, according to alignment strategy.

## Abbreviations

The following abbreviations are used in this manuscript:

KOA	Knee Osteoarthritis
TKA	Total Knee Arthroplasty
MA	Mechanical Alignment
KA	Kinematic Alignment
HKA	Hip–knee–ankle
|HKA − 180°|	Absolute HKA Deviation from the Neutral Mechanical Axis
MAD	Mechanical-Axis Deviation
|MAD|	Absolute Mechanical-Axis Deviation
KOOS	Knee Injury and Osteoarthritis Outcome Score
WOMAC	Western Ontario and McMaster Universities Osteoarthritis Index
KSS	Knee Society Score
ANCOVA	Analysis of Covariance
ASA	American Society of Anesthesiologists
BMI	Body Mass Index
CIs	Confidence Intervals
CPAK	Coronal Plane Alignment of the Knee
FDR	False Discovery Rate
ICCs	Intraclass Correlation Coefficients
KL	Kellgren–Lawrence
LDFA	Lateral Distal Femoral Angle
MCID	Minimal Clinically Important Difference
mPTA	Medial Proximal Tibial Angle
JLO	Joint-Line Obliquity
SD	Standard Deviation
PJI	Periprosthetic Joint Infection
PS	Posterior-Stabilized
ACL	Anterior Cruciate Ligament
PCL	Posterior Cruciate Ligament
