# Supplementary material for: Mechanical Versus Kinematic Alignment in Total Knee Arthroplasty: Relationship Between Postoperative Alignment and Short-Term Functional Outcomes
Source: Diagnostics (Basel). 2026 Oct 22;16(21):3446. doi: 10.3390/diagnostics16213446 (PMC13762007; doi:10.3390/diagnostics16213446)
Supplement: Supplementary file 1 [file diagnostics-16-03446-s001.zip › diagnostics-4615059-supplementary.pdf]

Supplementary Table S1. Domain-specific KOOS scores before and after total knee arthroplasty, according to alignment strategy

KOOS domain	MA preoperative (n = 51)	MA postoperative (n = 51)	KA preoperative (n = 69)	KA postoperative (n = 69)
Pain	44.08 ± 11.46	81.99 ± 14.93	49.34 ± 11.90	88.11 ± 11.34
Symptoms	41.64 ± 11.80	80.06 ± 15.72	46.78 ± 11.41	86.65 ± 11.34
Activities of Daily Living	47.41 ± 12.82	84.74 ± 13.58	52.42 ± 11.38	90.76 ± 9.68
Sport and Recreation	24.76 ± 12.06	65.40 ± 18.72	29.36 ± 12.44	73.43 ± 17.36
Knee-related Quality of Life	35.04 ± 11.15	73.40 ± 17.06	39.57 ± 12.01	81.25 ± 13.95

Note: Data are presented as mean ± standard deviation. KOOS, Knee injury and Osteoarthritis Outcome Score; MA, mechanical alignment; KA, kinematic alignment. Each KOOS domain is scored from 0 to 100, with higher scores indicating better knee-related outcomes. The five domains are presented separately to support interpretation of the study-defined composite KOOS score used in the main analyses.
